# Optimized and accelerated ^19^F‐MRI of inhaled perfluoropropane to assess regional pulmonary ventilation

**DOI:** 10.1002/mrm.27805

**Published:** 2019-05-17

**Authors:** Mary A. Neal, Benjamin J. Pippard, Kieren G. Hollingsworth, Adam Maunder, Prosenjit Dutta, A. John Simpson, Andrew M. Blamire, James M. Wild, Peter E. Thelwall

**Affiliations:** ^1^ Institute of Cellular Medicine Newcastle University Newcastle upon Tyne United Kingdom; ^2^ Newcastle Magnetic Resonance Centre, Campus for Ageing and Vitality Newcastle University Newcastle upon Tyne United Kingdom; ^3^ Newcastle upon Tyne Hospitals NHS Foundation Trust Newcastle upon Tyne United Kingdom; ^4^ POLARIS, Academic Unit of Radiology University of Sheffield, Royal Hallamshire Hospital Sheffield United Kingdom

**Keywords:** fluorocarbon gas, lung, perfluoropropane, pulmonary MRI, ventilation

## Abstract

**Purpose:**

To accelerate ^19^F‐MR imaging of inhaled perfluoropropane using compressed sensing methods, and to optimize critical scan acquisition parameters for assessment of lung ventilation properties.

**Methods:**

Simulations were performed to determine optimal acquisition parameters for maximal perfluoropropane signal‐to‐noise ratio (SNR) in human lungs for a spoiled gradient echo sequence. Optimized parameters were subsequently employed for ^19^F‐MRI of inhaled perfluoropropane in a cohort of 11 healthy participants using a 3.0 T scanner. The impact of 1.8×, 2.4×, and 3.0× undersampling ratios on ^19^F‐MRI acquisitions was evaluated, using both retrospective and prospective compressed sensing methods.

**Results:**

3D spoiled gradient echo ^19^F‐MR ventilation images were acquired at 1‐cm isotropic resolution within a single breath hold. Mean SNR was 11.7 ± 4.1 for scans acquired within a single breath hold (duration = 18 s). Acquisition of ^19^F‐MRI scans at shorter scan durations (4.5 s) was also demonstrated as feasible. Application of both retrospective (n = 8) and prospective (n = 3) compressed sensing methods demonstrated that 1.8× acceleration had negligible impact on qualitative image appearance, with no statistically significant change in measured lung ventilated volume. Acceleration factors of 2.4× and 3.0× resulted in increasing differences between fully sampled and undersampled datasets.

**Conclusion:**

This study demonstrates methods for determining optimal acquisition parameters for ^19^F‐MRI of inhaled perfluoropropane and shows significant reduction in scan acquisition times (and thus participant breath hold duration) by use of compressed sensing.

## 1 INTRODUCTION

Respiratory diseases are a leading cause of morbidity and mortality worldwide.^1^ Clinically, computed tomography (CT) and nuclear medicine techniques (e.g. planar scintigraphy and single photon emission computed tomography) facilitate assessment of both structural and functional properties of the lungs and are used routinely to aid diagnosis and monitoring of treatment response. However, these methods are limited by their reliance on ionizing radiation, restricting longitudinal or serial use.

Magnetic resonance imaging is increasingly recognized as a potential radiation‐free approach to the investigation of pulmonary disease. Specifically, hyperpolarized gas MRI is well established in research settings, permitting the study of regional ventilation across a variety of respiratory pathologies,^2^, ^3^, ^4^ yet the requirement for gas polarization equipment and expertise presents potential barriers to routine clinical application.

^19^F‐MRI of inhaled perfluoropropane is an emerging method for assessment of ventilation properties in humans.^5^, ^6^ This technique uses an inert, thermally polarized gas with multiple chemically equivalent ^19^F nuclei. The short in vivo T_1_ relaxation time of perfluoropropane (~12 ms at 3.0 T)^5^ permits short repetition time (TR), allowing a high degree of signal averaging and thus image acquisition without the need for hyperpolarization. Previous studies have demonstrated the feasibility of this approach to assess regional gas distribution^5^, ^6^ and washout dynamics^7^ in the lungs of healthy volunteers and patients with respiratory disease. These human studies have built upon comprehensive preclinical^8^, ^9^, ^10^, ^11^, ^12^ and ex vivo^13^ studies that demonstrated the technical approach and characterized physical and MR properties of in vivo fluorocarbon gases. Nonetheless, MRI of inhaled perfluoropropane remains challenging, largely driven by its short in vivo T_2_^*^ relaxation properties (~2 ms at 3.0 T).^5^

As with all MRI techniques, acquisition parameter choice fundamentally impacts scan efficiency and resultant SNR. Understanding the interdependency of scan acquisition parameters is therefore central to maximizing performance of this methodology. Compressed sensing (CS) methods can further exploit the sparsity of MR images under mathematical transformation,^14^ reducing the amount of raw data acquired for a given matrix size while preserving image quality and content.^15^, ^16^ This has potential substantially to reduce breath hold duration,^17^ which may be invaluable for assessing patients with compromised ventilation by reducing scan times or improving spatial resolution. Compressed sensing has shown utility in hyperpolarized ^3^He and ^129^Xe lung imaging,^18^, ^19^ where there is intrinsically high SNR. The degree to which undersampling and compressed sensing reconstruction can be applied with acceptable image fidelity is highly dependent on SNR and phase encoding matrix size.^20^ ^19^F‐MR ventilation imaging is characterized by lower SNR and smaller phase‐encoding matrices. Acceleration factors and phase encoding patterns validated for ^3^He‐MRI and ^129^Xe‐MRI are unlikely to be acceptable for ^19^F imaging. This work evaluates CS for ^19^F‐MRI of inhaled perfluoropropane for the first time.

In this study we assessed the impact of critical image acquisition parameters on spoiled gradient echo (SPGR) scan performance by calculating the interdependent effects of bandwidth (*BW*), excitation pulse amplitude and flip angle (B_1_, θ), and repetition time (TR) on SNR of ^19^F‐MRI scans of inhaled perfluoropropane. Additionally, we compared image SNR and measured lung ventilated volume and ventilation defect percentage (VDP) calculated from retrospectively and prospectively accelerated scans compared to fully sampled acquisitions to gauge utility of accelerated ^19^F‐MR ventilation imaging.

## 2 METHODS

### 2.1 ^19^F‐MRI sequence optimization

The standard equation for the signal intensity elicited by a SPGR sequence^21^ was modified to calculate the maximal SNR achievable within a defined scan duration, *SNR_SPGR_* (Equation 1). Simulations were then performed using the modified equation to assess variation in *SNR_SPGR_* with change in acquisition parameters, and to determine the parameters necessary for optimal *SNR_SPGR_*. The total scan time (*T_scan_*) was kept at an arbitrary constant, permitting assessment of scan performance variability within the finite scan time imposed by breath hold acquisitions (e.g. <20 s).

(1) $$SNR_{\mathrm{SPGR}}\propto FOV_{x}FOV_{y}FOV_{z}\sqrt{\frac{T_{\mathrm{scan}}}{N_{x}^{2}N_{y}^{2}N_{z}^{2}TRBW}}\frac{\left(1-e^{-TR/T_{1}}\right)\sin\theta }{\left(1-e^{-TR/T_{1}}\cos\theta \right)}e^{-TE_{\min}/T_{2}^{*}}$$

where *N_x_*, *N_y_*, and *N_z_* = number of frequency, and phase and partition encoding steps in each dimension; *BW* = receiver bandwidth (Hz/pixel); *T_scan_* = total scan time (s); *TE_min_* = minimum achievable echo time (s); T_2_^*^ = transverse relaxation time (s); T_1_ = longitudinal relaxation time (s); *FOV_x,y,z_* = field of view (mm); and TR = repetition time (s).

The scan acquisition parameters in Equation 1 are interdependent, such that adjusting one parameter to increase scan performance can have a subsequent impact on other parameters, with potential to reduce or negate SNR gains. For example, *TR_min_* is dependent on *TE_min_*, which in turn is dependent on bandwidth, matrix size, and radiofrequency (RF) pulse and gradient properties.

*SNR_SPGR_* was calculated for flip angles (θ) between 0 and 90 degrees, and for *BW* between 0 and 1500 Hz/pixel. The minimum echo time (*TE_min_*) and repetition time (*TR_min_*) were calculated as the minimum achievable on our scanner hardware based on RF pulse durations for B_1_ amplitudes between 0 and 10 µT, in accordance with scanner gradient rise times and amplitudes, over the ranges of flip angle, bandwidth, and B_1_ amplitude assessed. *TR_min_* was further bound by IEC 60601‐2‐33 specific absorption rate (SAR) limits^22^ applied to the ^19^F birdcage chest coil (Rapid Biomedical, Rimpar, Germany) in use at our center. The TR was therefore extended (i) to maintain whole‐body SAR limits under normal (2 W/kg) and first‐level (4 W/kg) operating modes and (ii) to maintain local torso limits under normal operating mode (10 W/kg).^22^

The field of view (*FOV_x,y,z_*) and matrix size (*N_x,y,z_*) used in our simulations were chosen to be representative of ^19^F‐MRI scans performed at our center (detailed later). Perfluoropropane T_1_ and T_2_^*^ were based on values observed in previously published human studies at 3.0 T (12.4 ms and 2.2 ms, respectively).^5^

The variation in *SNR_SPGR_* with change in θ, *BW* and TR was determined for a nonselective block RF pulse with B_1_ amplitude of 4 μT, corresponding to the B_1_ used in our human studies (outlined later). Acquisition parameters for optimal *SNR_SPGR_* were subsequently determined. The impact of changing B_1_ amplitude on optimal *SNR_SPGR_* was also calculated over a B_1_ range of 0 to 10 μT.

### 2.2 ^19^F‐MRI of inhaled perfluoropropane in healthy volunteers

Ethical approval for this study was granted by the Newcastle and North Tyneside 2 Research Ethics Committee and the NHS Health Research Authority. A total of 11 healthy volunteers (3 males, aged 25 to 46, mean = 33; 8 females, aged 24 to 63, mean = 34) were screened for study eligibility across two research sites (Newcastle and Sheffield) and provided written informed consent to participate. All participants were nonsmokers in good health with no history of respiratory disease and no contraindications to MRI. Body weights were maintained within lower and upper weight limits specified by the RF coil manufacturer (50 kg and 100 kg, respectively).

Participants were invited to attend a single MRI scan session at one of the two research centers, during which they underwent conventional ^1^H‐MRI and ^19^F‐MRI scans, performed using a Philips Achieva (Newcastle) or Philips Ingenia (Sheffield) 3.0 T MRI scanner and chest birdcage ^19^F/^1^H coil (Rapid Biomedical, Rimpar, Germany). ^1^H images were acquired using a multislice SPGR sequence (TE = 2.3 ms; TR = 5.1 ms; FOV = 450 × 450 × 300 mm; resolution = 192 × 96 × 30, reconstructed to 256 × 256 × 30; BW = 450 Hz/pixel; flip angle = 40°; scan duration = 15 s) with standard elliptical window^21^ after instructing participants to perform a breath hold at total lung capacity.

Participants were then asked to inhale a clinical grade 79% perfluoropropane/21% oxygen gas mixture (BOC Special Products, Guilford, United Kingdom), which involved up to five inhalation sessions during the MRI scan session, where one (retrospective scan acceleration measurement) or two (prospective scan acceleration measurements) of the inhalation sessions were used to generate data for the study reported here. The remaining inhalation sessions were employed for other method development or research study purposes. Each inhalation session lasted less than 1 min, comprising three deep breaths of the gas via a nonrebreathe valve and mouthpiece (Hans Rudolf, Shawnee, KS), followed by a breath hold at total lung capacity. ^19^F‐MR images were subsequently acquired using a 3D SPGR sequence (1 cm isotropic resolution; FOV = 40 × 32 × 25 cm, zero‐filled to a reconstruction resolution of 0.36 × 0.36 × 1 cm; matrix = 112 × 90 × 25). The optimized acquisition parameters derived from simulation studies were TE = 1.7 ms; TR = 7.5 ms; BW = 500 Hz/pixel; flip angle = 50°, nonselective block RF pulse with a B_1_ amplitude of 4 μT. The duration of a single 3D gradient echo image was 4.5 s. Scans were acquired with four signal averages in total (scan duration = 18 s).

### 2.3 Compressed sensing for accelerated ^19^F‐MRI

**Figure 1:**
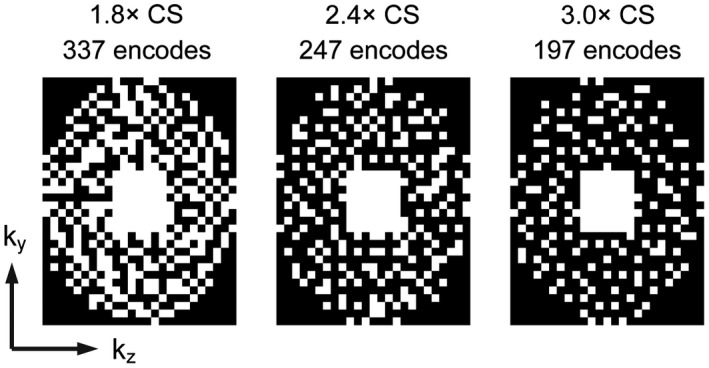
Phase encoding undersampling schemes shown for 1.8×, 2.4×, and 3.0× CS schemes; CS, compressed sensing

Fully sampled k‐space data were acquired from eight participants in an 18‐s scan (number of signal averages, NSA = 4). The data were retrospectively undersampled in both phase encoding directions (i.e. ky and kz, corresponding to physical directions right‐left and anterior‐posterior respectively, 32 × 25 matrix). The undersampling schemes were designed to create an incoherent sampling pattern possible with standard Fourier transformation using a uniform density Poisson disc with a fully sampled center.^23^ Although our previous work has used variable density Poisson discs to reflect the underlying distribution of k‐space,^17^ the small matrix size used for this application provided too few samples at the edge of k‐space. Three degrees of undersampling were considered (1.8×, 2.4×, and 3.0×, sampling patterns shown in Figure 1), which would lead to breath holding times of 10 s, 7.5 s, and 6 s, respectively, with four averages. The sampling patterns were executed within a standard elliptical window for 3D gradient echo and there was full sampling of a central 8 × 7 region. A full acquisition with elliptical window has 592 phase encodes. Where undersampled data were acquired prospectively, a custom pulse sequence, which could faithfully reproduce the undersampled phase encode patterns to reduce the acquisition time, was used.

The incoherently undersampled data were reconstructed using an L1‐ESPIRiT algorithm with wavelet regularization as described in^17^ performed by minimizing:

$$\|y_{i}{-DFm\|}_{2}^{2}+\lambda {\left\|\Psi m\right\|}_{1}$$

where *y_i_* is the acquired data, *m* is the reconstructed image space to find, *F* is a Fourier transform operator, and *D* is an operator that selects only those locations where data have been acquired (to match *y_i_*).^24^ The Daubechies‐4 wavelet was used as the sparsifying transform, Ψ, and the weighting between the fit to the data and the sparsity of *m* in the wavelet domain was provided by λ. While there are many possible transforms that can sparsify MRI data, the Daubechies‐4 wavelet is computationally undemanding and has proven excellent performance across a range of compressed sensing applications in MRI.^15^, ^17^, ^24^, ^25^ Randomized shifting of the wavelet transform was used to approximate translation‐invariant wavelets and prevent the appearance of structured artefacts within the reconstructed images.^25^, ^26^ Fifty iterations were used to ensure convergence of the solution. In order to determine the optimal value of the wavelet weighting parameter, λ, the retrospectively sampled raw data were reconstructed with several different values of λ and the root mean square error was calculated between the fully sampled and undersampled data, using a signal intensity threshold to ensure only lung signals were evaluated. Lambda (λ) was chosen to minimize the root mean square error and an optimal value of 0.05 was used throughout.

The fully sampled and retrospective accelerated reconstructions were compared by subtraction and the apparent SNR measured. The SNR achieveable in 18 s (NSA = 4) of ^19^F‐MR images was determined for all participants. Regions of interest 4 cm in diameter were placed in the center of the left lung (signal) and below the lung (noise) and SNR calculated using in‐house software developed in Matlab (Mathworks Inc., Natick, MA) using the equation SNR = 0.66 × mean signal amplitude/standard deviation of the noise, where 0.66 is the Rayleigh distribution correction.^27^

Images acquired using optimized ^19^F scan parameters were also reconstructed from the first signal average (4.5 s) of the dataset.

A prospectively undersampled 3D gradient echo scan was acquired from a further three participants using the 1.8× acceleration scheme and NSA = 4 (scan duration = 10 s), using a custom pulse sequence to perform the selective phase encodings for the undersampling scheme in Figure 1,^23^ in addition to a fully sampled dataset acquired in a separate breath hold.

### 2.4 Measurement of lung ventilated volume and ventilation defect percent

The ventilated volumes of the fully sampled and undersampled 1.8× CS data were measured using an open‐source semiautomated 3D segmentation tool (ITK‐SNAP).^28^ A signal threshold of 3 SD below the mean signal from each image was adopted for the purpose of calculating ventilated lung volumes (L) of inhaled perfluoropropane. The trachea and main bronchi were excluded from the analysis through manual segmentation. A paired *t* test was conducted to assess for significant change in measured ventilated volumes between the fully sampled and retrospectively 1.8× undersampled images. Measurements of lung VDP were calculated as the difference between total lung volume and lung ventilated volume, where total and ventilated lung volume were calculated from coregistered ^1^H‐MRI and ^19^F‐MRI datasets by a semiautomated approach using in‐house software developed in Matlab.

## 3 RESULTS

**Figure 2:**
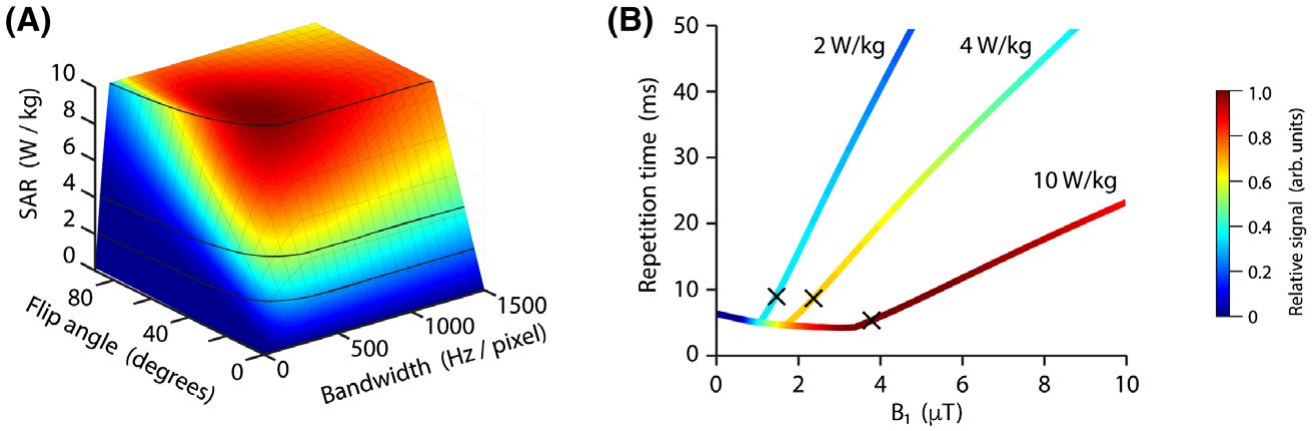
A, Relative scan SNR (colorscale) achievable in a fixed acquisition time over a range of flip angles and acquisition bandwidths for a 3D SPGR acquisition sequence. Scan SAR (shown in the vertical axis) was limited to local and torso SAR limits of 10 W/kg by extension of scan repetition time. SAR isolines highlight positions of 2, 4, and 10 W/kg. B, Relative scan SNR (colorscale) achievable over a range of excitation B_1_ values (*x*‐axis) with optimal (shortest achievable) TR for SAR = 2, 4, and 10 W/kg. The point at which maximum SNR is reached is marked (×) for each SAR level; SAR, specific absorption rate; SNR, signal‐to‐noise ratio; SPGR, spoiled gradient echo; TR, repetition time

Figure 2 shows the results of simulations investigating the relationship between SPGR acquisition parameters and ^19^F‐MRI scan SNR performance. Figure 2A shows the impact of flip angle and acquisition bandwidth on SNR (shown in colorscale) and SAR (z‐axis) for scans performed with a nominal B_1_ of 4 μT. Maximal SNR is observed at an acquisition bandwidth of 500 Hz/pixel and a flip angle of 40°. The data demonstrate a sharp decrease of SNR with reduction of flip angle slightly below optimal, but a less marked reduction with flip angle increasing above the theoretical optimum. Thus, the use of a flip angle slightly higher than the predicted optimal value was considered beneficial for our in vivo applications, where B_1_ inhomogeneity is present. Figure 2B shows the impact of B_1_ amplitude on optimal values of TR, where relative SNR is shown in colorscale and plots are drawn for three SAR limits (2, 4, and 10 W/kg, respectively). Scan performance shows the greatest dependence on B_1_ amplitude below 2 μT, with diminishing SNR gains for B_1_ amplitudes above this threshold. The impact of SAR limits on acquisition parameters is apparent as an abrupt increase in TR and flip angle above a threshold B_1_ amplitude, as scan duty cycle is reduced to accommodate elevated SAR arising from higher B_1_.

**Figure 3:**
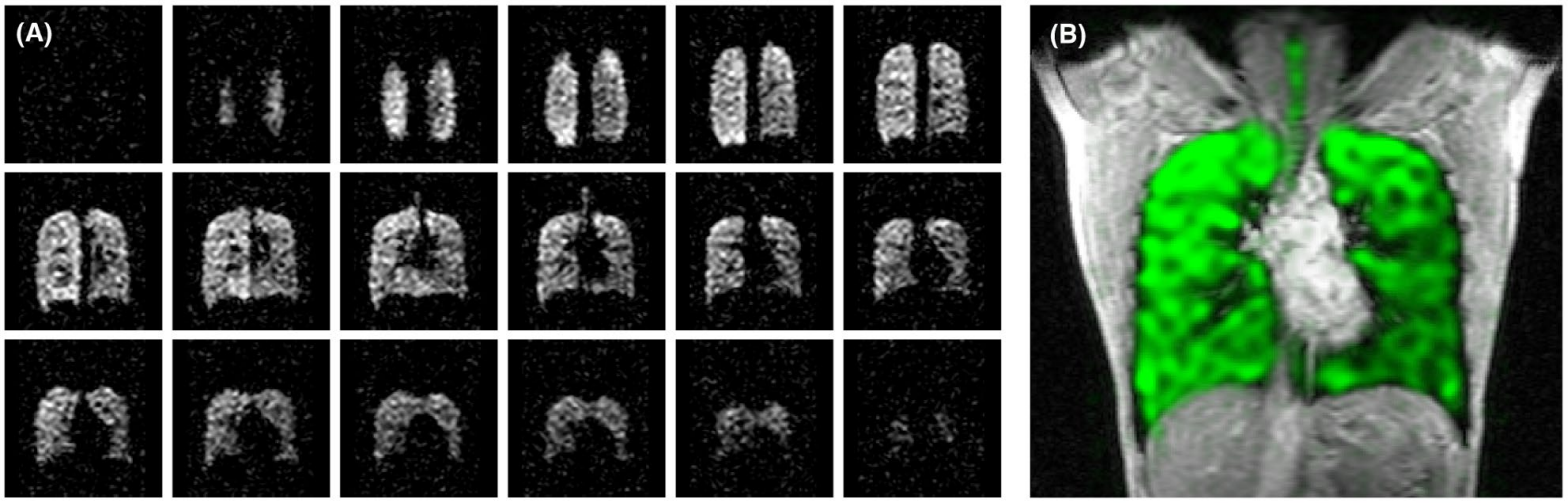
A, Coronal slices from a 3D ^19^F SPGR acquisition of 4.5‐s duration, acquired from a healthy volunteer. Mean SNR was 7.7. B, Healthy volunteer lung image generated from combined single slices of 3D ^19^F and ^1^H SPGR scans; SNR, signal‐to‐noise ratio; SPGR, spoiled gradient echo

Figure 3A demonstrates typical ^19^F‐MR images from one participant, acquired in 4.5 s (NSA = 1) using a 3D SPGR scan with optimized acquisition parameters. The B_1_ amplitude was 4 μT, with power deposition kept within local torso limits (10 W/kg) as specified by IEC 60601‐2‐33.^22^ Mean SNR was 7.7. A single slice from the 3D ^19^F‐MRI dataset, superimposed on an anatomical ^1^H image, is shown in Figure 3B.

**Figure 4:**
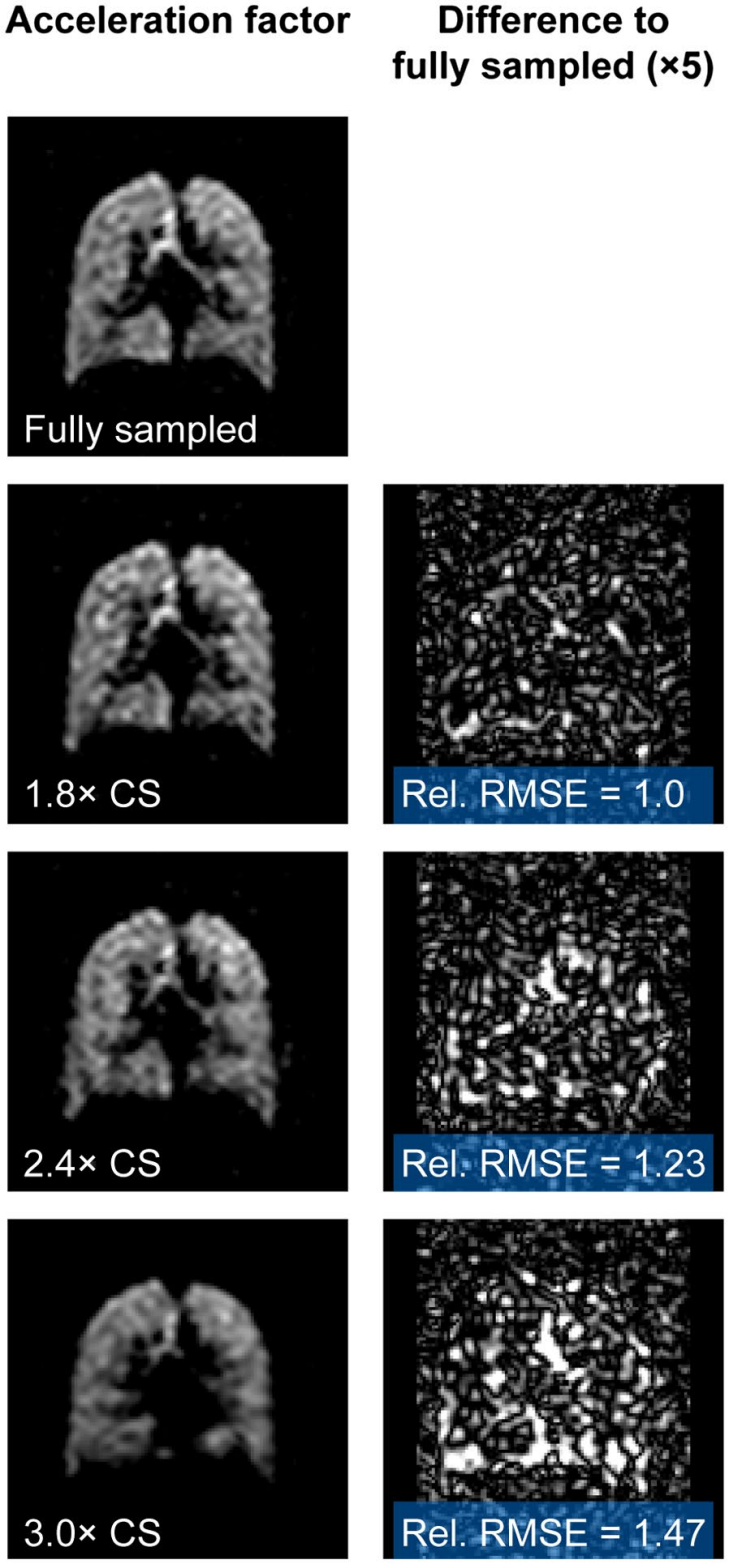
^19^F lung images reconstructed from NSA = 4 fully sampled healthy volunteer scan data and from retrospective undersampled reconstructions of the same dataset (undersampling of 1.8×, 2.4×, and 3.0×), and images of the difference between CS and fully sampled images shown at 5× vertical scale. Relative root mean square error provides an index of similarity between fully sampled and undersampled images, scaled to the 1.8× dataset; CS, compressed sensing; NSA, number of signal averages

**Figure 5:**
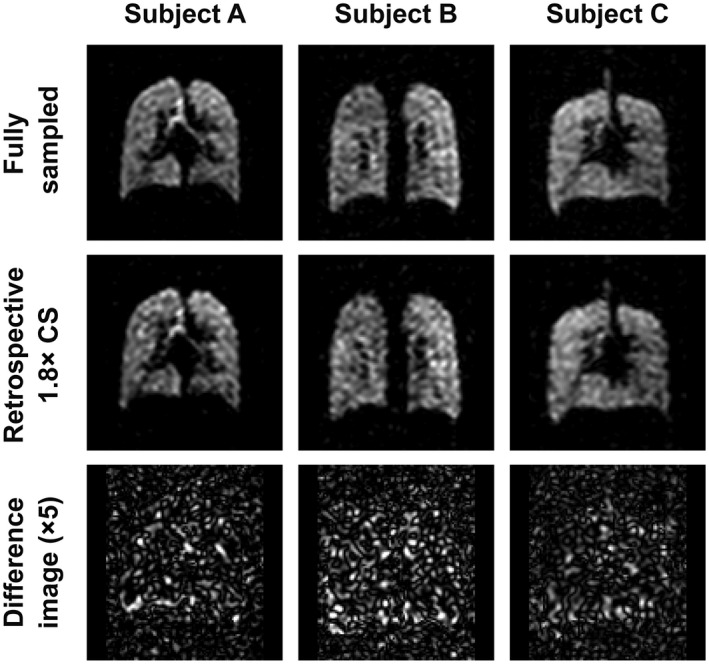
Coronal slices from 3D ^19^F acquisitions (NSA = 4) from three representative participants, showing fully sampled acquisitions (*top*), images reconstructed from 1.8× retrospective undersampling (*center*), and difference images between fully sampled and undersampled reconstructions at 5× vertical scale (*bottom*); NSA, number of signal averages

Figure 4 shows a single coronal slice from a fully sampled lung volume (NSA = 4, scan duration = 18 s) in one healthy participant, alongside comparative images resulting from retrospectively applied compressed sensing (undersampling ratios of 1.8×, 2.4×, and 3.0×). Subtraction images demonstrate that the 1.8× undersampled dataset closely matches the fully sampled dataset. Greater differences can be observed between the fully sampled and undersampled datasets as the acceleration factor increases beyond 1.8×, with increasing root mean square error between the fully sampled and accelerated image pairs. The root mean square error values in Figure 4 have been expressed as multiples of that for the 1.8× undersampling for ease of comparison. Similar results were obtained across the eight participants (SNR 10.8 ± 3.8 and 11.2 ± 3.7 for fully and retrospectively undersampled datasets, respectively), such that the 1.8× undersampling ratio was considered most appropriate for acceleration purposes. Single coronal slices from 3D datasets from three representative participants are shown in Figure 5, with the 1.8× undersampled datasets demonstrating negligible differences when compared to the fully sampled datasets. Table 1 shows SNR, ventilated volume, and VDP measurements calculated from fully sampled and retrospectively accelerated datasets for each of the eight participants in the group. Ventilated volume and VDP values calculated from retrospectively accelerated datasets were close to those calculated from fully sampled datasets, though the majority of ventilated volume measurements calculated from 1.8× undersampled datasets were slightly lower than those calculated from fully sampled data, and corresponding VDP measurements slightly higher in undersampled datasets compared to fully sampled data. These differences were not statistically significant.

**Table 1:** Signal‐to‐noise ratio, lung ventilated volume, and ventilation percentage defect measurements calculated from fully‐sampled and retrospectively accelerated scan datasets from eight healthy volunteer participants

Participant	Fully sampled			Retrospectively accelerated		
	SNR	VV / L	VDP / %	SNR	VV / L	VDP / %
A	13.5	4.67	0.6	13.8	4.49	1.1
B	5.8	4.70	1.5	6.4	4.35	1.5
C	15.9	5.65	0.5	17.0	5.47	2.7
D	8.6	7.76	2.1	7.9	7.26	3.7
E	7.9	4.36	2.4	8.9	3.92	2.6
F	7.6	6.33	1.7	9.1	5.89	1.7
G	14.3	3.82	2.9	14.4	3.48	2.3
H	13.1	6.27	0.4	12.5	6.07	0.7
Mean	10.8	5.45	1.5	11.3	5.12	2.0

Abbreviations: SNR, signal‐to‐noise ratio; VDP, ventilation defect percentage; VV, lung ventilated volume.

**Figure 6:**
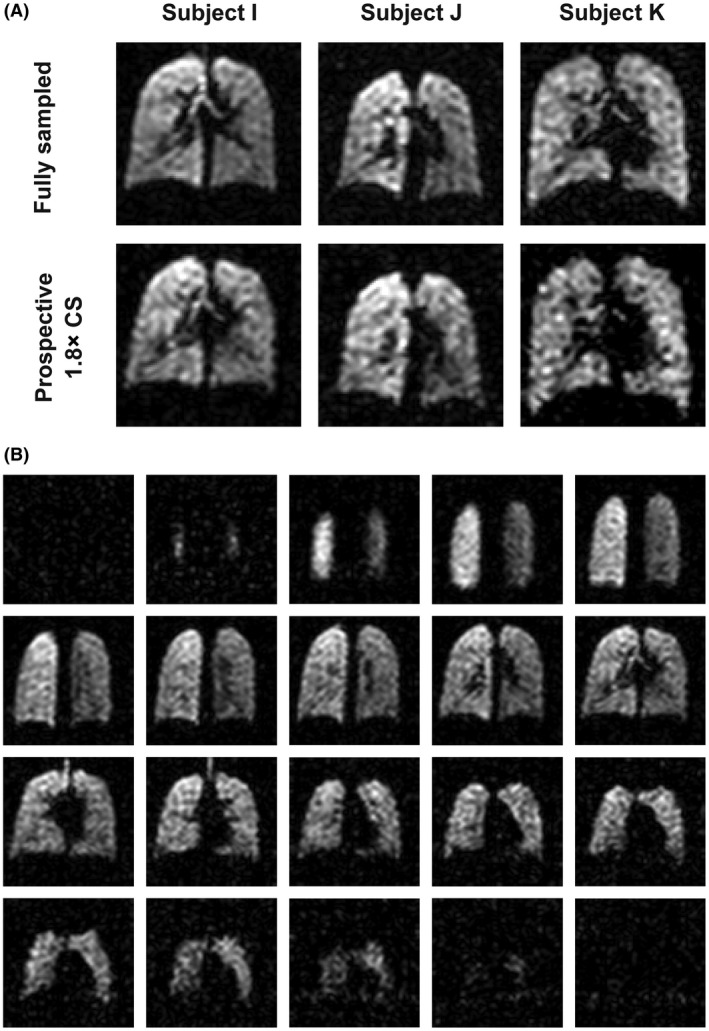
A, Coronal slices from 3D fully sampled and prospectively 1.8× accelerated scans (NSA = 4), acquired from three healthy volunteers in separate breath holds. Difference images are not shown because of lack of registration between images resulting from minor difference in lung inflation levels between breath holds). B, A 3D ^19^F‐MRI scan acquired with 1.8× prospective acceleration (NSA = 4) in a scan of 10‐s duration, showing whole‐lung coverage at 1‐cm isotropic resolution; NSA, number of signal averages

Figure 6A shows single coronal slices from prospective 1.8× accelerated 3D acquisitions from three participants, alongside corresponding fully sampled datasets acquired in a separate breath hold. The accelerated datasets have comparable visual appearance to the undersampled datasets. Subtraction images between the fully sampled and accelerated datasets were not generated as data were acquired in separate breath holds and thus lung spatial alignment and inflation are not matched. The SNR of prospectively acquired undersampled datasets was 13.4 ± 4.0, comparable to the fully sampled datasets in these participants, 14.1 ± 5.0. Figure 6B shows an entire prospectively accelerated ^19^F‐MRI dataset from one of the three participants, demonstrating whole‐lung coverage in a 10‐s duration scan. Table 2 shows SNR, ventilated volume, and VDP measurements calculated from fully sampled and prospectively accelerated datasets for each of the three participants in the group.

**Table 2:** Signal to noise ratio, lung ventilated volume, and ventilation defect percentage measurements calculated from fully sampled and prospectively accelerated scan datasets from three healthy volunteer participants

Participant	Fully sampled			Prospectively accelerated		
	SNR	VV/L	VDP/%	SNR	VV/L	VDP/%
I	10.0	5.27	1.3	9.9	5.13	1.9
J	19.7	5.75	0.8	17.7	5.56	1.2
K	12.5	4.33	0.3	12.6	4.29	2.7
Mean	14.1	5.12	0.8	13.4	4.99	1.9

Abbreviations: SNR, signal‐to‐noise ratio;VDP, ventilation defect percentage; VV, lung ventilated volume.

**Figure 7:**
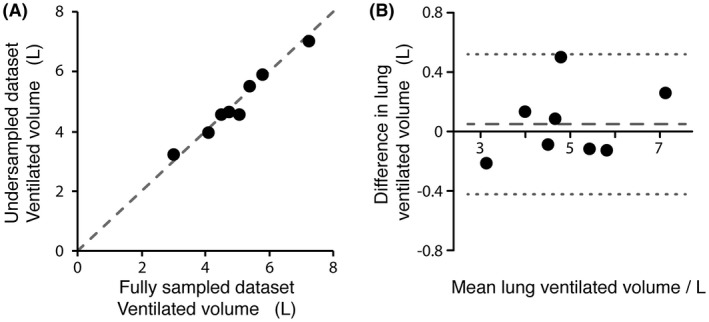
A, correlation and B, Bland‐Altman plots showing comparison of ventilated volumes measured calculated from fully sampled and 1.8× retrospectively undersampled datasets. The correlation plot shows an isoline between measurements. The calculated correlation coefficient was 0.982. The Bland‐Altman plot shows estimated bias (0.05 L, — — —) and 95% limits of agreement (±0.47 L, · · ·)

A paired‐samples *t* test demonstrated no significant difference in lung ventilated volume measurements made from eight fully sampled (mean = 5.0 L, SD = 1.2 L) and 1.8× retrospectively undersampled (mean = 4.9 L, SD = 1.1 L) lung ^19^F‐MRI datasets (*p* = .59). The lung ventilated volume measurements calculated from 1.8× prospectively undersampled acquisitions in three participants (mean = 5.0 L, SD = 0.6 L) closely matched those from the fully sampled acquisitions in these participants (mean = 5.1 L, SD = 0.7 L) and the difference in volume between the two groups was not statistically significant (*p* = .11). Figure 7 shows (A) correlation and (B) Bland‐Altman plots of ventilated volume measured from fully sampled and 1.8× undersampled datasets for the eight participants. The bias between undersampled and fully sampled datasets by Bland‐Altman analysis (0.05 L) was not statistically significant.

**Figure 8:**
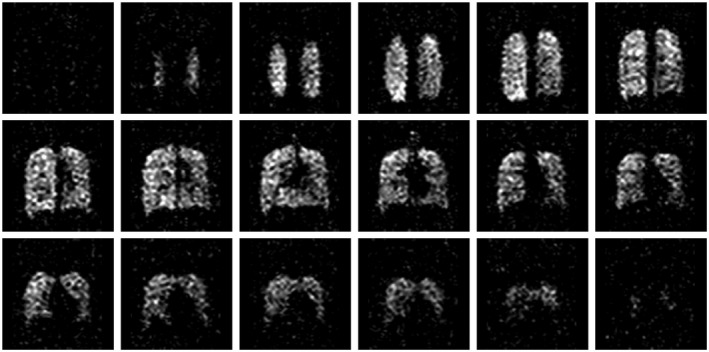
Retrospective 1.8× undersampling of the NSA = 1 dataset shown in Figure 3 (shown with the same window and level scaling as Figure 3), corresponding to a 2.5‐s acquisition duration for a prospective acquisition; NSA, number of signal averages

Figure 8 shows a 3D image dataset reconstructed from a single average (NSA = 1) of the dataset used to generate Figure 3, with 1.8× retrospective undersampling. The scan duration of a corresponding prospective acquisition with this undersampling scheme would be 2.5 s. The images show good visual correspondence with the fully sampled images shown in Figure 3.

## 4 DISCUSSION

We have modeled ^19^F‐MRI scan performance to optimize imaging of inhaled perfluoropropane with a SPGR pulse sequence at 3.0 T and demonstrated the potential for ^19^F‐MRI scan acceleration through compressed sensing methods in a group of healthy participants. The interdependent relationship between MR acquisition parameters is particularly marked for perfluoropropane imaging because of inherently short relaxation rates (T_1_ and T_2_^*^) and operation at or near SAR limit boundaries. Our SNR calculations enable determination of optimal acquisition parameter values based on hardware performance, providing a robust and comprehensive understanding of their interdependence.

The short T_1_ of perfluoropropane has the advantage of permitting short TR, and thus a high degree of signal averaging, with minimum TR determined by the SAR limits of our hardware configuration. Our calculations show that short TR (<10 ms) and large flip angles (40° to 50°) are required for maximal scan efficiency, and that the short T_2_^*^ of perfluoropropane causes acquisition bandwidth and excitation pulse duration to have a strong impact on scan performance. A reduction in RF pulse duration through increased B_1_ amplitude offers diminishing returns to scan efficiency above approximately 2 μT on our hardware configuration. This effect is significant as scanner multinuclear (nonproton) RF amplifiers typically have considerably lower power output than their ^1^H counterparts, such that the maximum achievable B_1_ may be modest for ^19^F torso coils.

^19^F‐MR images of inhaled perfluoropropane acquired from our group of healthy participants showed a mean SNR of 11.7 ± 4.1 for a scan performed within a single breath hold (18 s, NSA = 4) with 1‐cm isotropic acquisition resolution. A direct SNR comparison of our scan performance with previously published ^19^F‐MRI studies is challenging as a result of differences in RF coil hardware, scanner field strength, and scan voxel sizes and of a lack of comprehensive information regarding choice of scan parameters. Nonetheless, the optimization approach we describe provides a framework to ensure maximal scan performance is achieved for a given hardware configuration. In addition, we have demonstrated that gains in temporal resolution are achievable through the use of compressed sensing methods. Specifically, the application of a 1.8× undersampling scheme and compressed sensing reconstruction is able to preserve image quality and apparent SNR. As well as improving temporal resolution in dynamic imaging, the acceleration offered by compressed sensing has potential to reduce breath hold duration (particularly significant for patients unable to comply with longer breath holds), or to improve SNR by enabling a greater degree of signal averaging during breath hold. The achievable acceleration for this ^19^F‐MRI protocol is, as expected, not as great as in previous hyperpolarized gas MRI studies,^18^, ^19^ reflecting the inherently lower signal generated by thermally polarized perfluoropropane and the relatively small phase‐encoding matrix used. Nonetheless, our data clearly demonstrate the utility of compressed sensing for ^19^F‐MRI of perfluoropropane, despite these limitations.

Our data show the majority of VDP values calculated from 1.8× undersampled datasets were slightly higher than those calculated from fully sampled data, though the difference in VDP between the two groups was not statistically significant. Scan acceleration introduced subtle signal intensity changes in ^19^F‐MRI scans that had a small effect on the position of the boundary between ventilated and nonventilated regions, manifest as a small decrease in lung ventilated volume. The VDP measurements of the prospectively accelerated group did not show a statistically significant difference from those of the retrospectively accelerated group (*t* test, *p* = .87). Future studies employing fully sampled and accelerated ^19^F‐MRI on patients with ventilation defects arising from respiratory disease will provide insight into the extent to which this effect might impact measurements made from a clinical cohort and allow testing of mitigation strategies if required (for example, alteration of region of interest threshold boundary based on degree of acceleration). Nonetheless, the difference in mean VDP between accelerated and fully sampled scans remains small (2.0% versus 1.5%, respectively).

Studies have demonstrated that non‐Cartesian acquisition techniques such as ultra short echotime (UTE) imaging^29^ hold value in ^19^F‐MRI of perfluoropropane as short echo times minimize T_2_^*^‐related signal losses. These approaches might also provide opportunities for higher acceleration factors. Further gains in scan performance may be achieved through the use of multichannel receive array coils, improving sensitivity through a smaller effective receive coil volume and enabling additional scan acceleration via parallel imaging methods. The efficacy of multichannel receive hardware for ^19^F‐MR imaging has already been successfully demonstrated in studies of patients with chronic obstructive pulmonary disease.^7^ The possibility of using this approach in combination with compressed sensing methods^14^ holds significant potential for additional scan acceleration and optimization.

The ability to breathe perfluoropropane with oxygen over a prolonged, dynamic image acquisition has recently shown promise in providing a quantitative measure of ventilation defects in patients with COPD.^7^ Such dynamic imaging has an advantage over hyperpolarized gas MRI in that the thermally polarized perfluoropropane exhibits scan signal intensity that is proportional solely to its concentration and relaxation properties within the lungs, whereas signal intensity of a hyperpolarized gas exhibits loss of polarization through RF‐mediated and T_1_‐mediated effects. The short duration of our optimized scan protocol may be suited to dynamic imaging.

^19^F‐MRI of inhaled perfluoropropane represents a nascent field, offering new opportunities for assessing pulmonary ventilation properties in both healthy volunteers and patients with respiratory disease. The optimization approach employed in our studies can be applied to different RF coil hardware configurations (including the combined use of array receive coils), different scanner manufacturers and models, and different scan acquisition methods to produce a tailored, optimized scan protocol. Improvements in scan optimization and acceleration offer considerable scope for future clinical application of this technique.
